# Hotels and Their Critics

**Published:** 1889-09-21

**Authors:** 


### "HOTELS AND THEIR CRITICS."

I have waited a month or so in the hope of someone accepting the challenge tendered in your able leader of the 20th ult., failing this, I must only conclude "that silence speaks consent," and my assertions hold their ground. However, I have to thank you for the publicity given, and if your columns can afford me space I crave a few lines in justification of my charges as well as in reply to your trenchant and rather ridiculing article. Had I anticipated that the puissant editorial pen would have directed its most barbed point against the most sensitive and least-protected part of my human environment—viz., mine honour—I should have furbished up my rusty weapons and come to the fray armed *cap a pie*, for a lethal weapon is this said editorial pen—truly mightier than the sword—but my sole object was to try and induce a remedy of gross defects by co-operation where individual effort is hopeless.

After a quarter of a century's experience of almost daily residence in hotels, varying in class from what is said to be "the most comfortable in Europe" to the humblest road-side inn, I thought myself fairly well qualified to venture an opinion, still I did not dare to enumerate simply personal experiences of the hundreds of hostelries at which I must have stayed. Neither did I formulate charges of dishonesty or extortion against a class of tradespeople who, I have reason to know, number in their community as upright and honourable men as any similar grade in this kingdom. My diatribe was solely directed against the *system*, of which, too frequently, the smaller hotel-keeper is the unfortunate victim; namely, the limited company hotel, in which an official retains the management, (be he called landlord, manager, maitre d'hotel, or anything you like) purely on condition he secures a dividend, pull baker, pull devil.

It is this system which is sapping the vitals of "mine host at mine inn," and replacing him by a frigid official indifferentism, which is well exemplified by those modern encaustic tiles which one reads in the vestibules of the grand limited, "Welcome the coming, speed the parting guest." Yes, more likely to break your neck than your fast any day.

On a subject, perhaps, more akin to your pages, I did not venture even to allude—viz., the insanitary conditions under which hotel *habitués* exist; and here let me add I am not commenting upon those imposing-looking certificates of eminent sanitary engineers, because such matters are beyond their scope. If cleanliness be next to godliness, both qualities are a long way outside hotels; but this divergence will most unwittingly draw "Experientia" into telling what his eyes have beheld, and these would really shock your readers too much; he pauses, and enumerates a few ordinary objectionables. Emptying contents of baths by means of hot-water cans provided for toilet purposes only, utilising utensils for similar objects, emptying contents of slop-pails into baths in bathrooms to save distance, dirty linen or household requisites being washed in said baths, washing water-bottles in slop-pails, sheets being sprinkled and pressed after visitor No. 1 to look fresh for No. 2, general indifference as to cleanliness of glasses, etc., used by invalids, carelessness as to protecting eatables, etc., from vermin, flies, etc., which gives rise to such delicacies as cockroaches in teapots, etc.

From one of the acknowledged authorities on cookery, I extract the following, which admirably embodies my contention:

"There is no beverage which is held in more universal esteem than good coffee, and none, in this country at least, which is obtained with greater difficulty. The coffee at hotels, etc., is so bad they reject it as too nauseous to be swallowed. A little national pride ought surely to prevent this, for to exact the full price of a good commodity and habitually to supply only trash for it is a commercial disgrace."—*Vide* Miss Acton.

In conclusion, Mr. Editor, I would remind you that as my letter was published purely *a discretione*, and therefore voluntarily, there is "the quip valiant" and "the retort courteous," but to attempt to give "the lie direct" to a correspondent, of whom you know nothing, and to insinuate cowardice is scarcely consistent with the spirit of fairplay of which you pose as so doughty a champion. Perhaps on a closer acquaintance you may find "Experientia" one who has always met an honourable adversary in the open, and one never desirous of sneaking behind a loop-holed defence, and certainly not now anxious to shelter beneath the editorial ægis, but "though all things may be possible, all things may not be expedient" for

August 26, 1889. Experientia Docet.

